# An Overview of Emphysematous Epididymo-Orchitis: A Systematic Review of Case Reports

**DOI:** 10.7759/cureus.38326

**Published:** 2023-04-30

**Authors:** Akram Bokhari, Hadi Aldarwish, Fatima Albladi, Abdulhakeem Almarzooq, Hatim Alqutayfi, Mohammed Alamer

**Affiliations:** 1 Department of Urology, University of Hail College of Medicine, Hail, SAU; 2 Department of Urology, Miami Cancer Institute, Florida, USA; 3 Department of Surgery, University of Hail College of Medicine, Hail, SAU; 4 Department of Surgery, King Faisal University College of Medicine, Al-Ahsa, SAU

**Keywords:** infection, gas shadow, epididymitis, epididymo-orchitis, emphysematous

## Abstract

Emphysematous epididymo-orchitis (EEO) is a rare but serious condition that involves the presence of gas within the tissues of the testicle and/or the epididymis. It is a medical emergency that can be life-threatening if left untreated. Management of this condition may involve a combination of antibiotics, surgical drainage, and supportive care. In March 2023, A systematic review of case reports was conducted to identify and examine cases of EEO. We used PubMed, ScienceDirect, and Google Scholar for a methodical search. Only seven out of 136 studies met our criteria for this review of case reports. However, this review discusses symptom presentation, imaging findings, complications, and possible management of EEO.

## Introduction and background

Emphysematous epididymo-orchitis (EEO) is a rare but serious condition that involves the presence of gas within the tissues of the testicle and/or the epididymis [1]. This condition is typically caused by bacterial infections and can result in severe pain, swelling, and inflammation in the affected area [2]. EEO is considered a medical emergency, as it can lead to significant morbidity and mortality if left untreated [3].

Several studies have highlighted the importance of early diagnosis and treatment of EEO. A previous study published found that delayed diagnosis and treatment of this condition were associated with a higher risk of testicular loss and mortality [4]. Also, another study emphasized the need for prompt surgical intervention to prevent these complications [5].

In this context, it is essential to recognize the clinical features and risk factors associated with EEO, and to ensure that patients receive timely and appropriate management. This may involve a combination of antibiotics, surgical drainage, and supportive care [6,7].

However, This article aims to review the EEO cases to improve comprehension of the presentation, clinical feature, diagnostic process, and outcome of this rare but potentially serious condition.

## Review

Materials and methods

In March 2023, following the Preferred Reporting Items for Systematic Reviews and Meta-Analyses (PRISMA) guidelines [8], we conducted a comprehensive review of case reports worldwide to identify and analyze cases of EEO. Our search included the following databases: PubMed, ScienceDirect, and Google Scholar.

Search Strategy

We employed the following search strategy on PubMed to identify relevant articles on EEO. Initially, we identified keywords related to the condition and then combined them with Medical Subject Headings (MeSH). The final search strategy was developed using the Medical Literature Analysis and Retrieval System Online (MEDLINE), with Boolean operators integrated as follows: "Epididymo-orchitis OR acute epididymo-orchitis OR acute epididymitis OR gas-forming epididymo-orchitis OR emphysematous epididymo-orchitis".

We used the keywords "Emphysematous epididymo-orchitis" to search for relevant articles on ScienceDirect and Google Scholar, in order to ensure that our study focused on the specified area of interest.

Inclusion and Exclusion Criteria

To thoroughly investigate relevant cases of EEO and its current management, we applied the criteria outlined in Table 1 during the data extraction process.

**Table 1:** List of inclusion and exclusion criteria

Criterion	Description
Criterion one	Fields of study related to medicine.
Criterion two	Review of reports with a publication date between the past 30 years (1993 - 2023).
Criterion three	Selection of case reports only.
Criterion four	Sorting especially for free full-text articles.

Results

**Figure 1:**
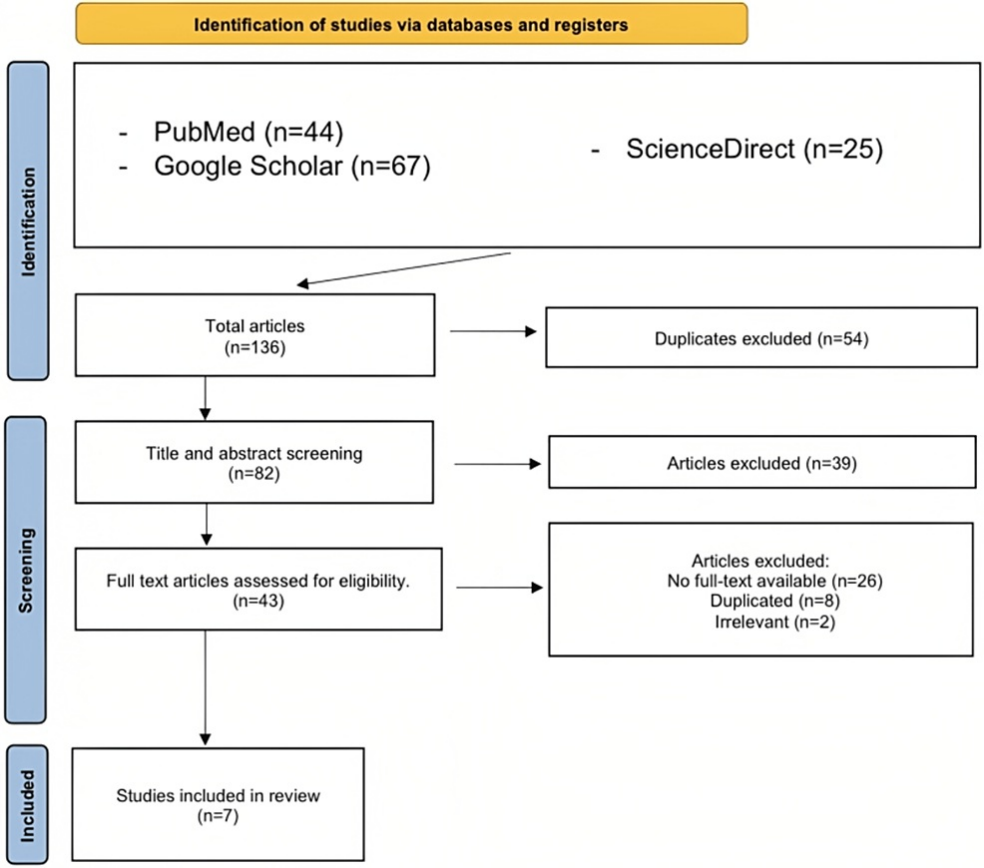
PRISMA flowchart PRISMA - Preferred Reporting Items for Systematic Reviews and Meta-Analyses References: [2,9-14]

Our search, using the methods described above, generated 136 publications from various databases. We used EndNote (Clarivate, London, United Kingdom) and manual comparison to identify and remove 54 duplicate publications from the initial investigation, leaving us with 82 articles for further evaluation. We conducted a thorough screening of the titles and abstracts of these articles to determine their relevance to our research topic. Subsequently, we assessed the quality of the remaining seven studies using the Joanna Briggs Institute (JBI) Critical Appraisal Checklist for Case Reports. After the application of the inclusion criteria, only seven case reports from different countries remained (Figure 1).

Table 2 provides a summary of the seven cases, outlining patient demographics, country of origin, comorbidities, patient presentation, microbiological diagnosis, and management plan.

**Table 2:** Overview of selected cases

Author	Year	Patient demographics	Country	Comorbidities	Presentation	Microbiological diagnosis	Management
Yen et al. [2]	2016	69-year-old	Taiwan	NA	Right side acute scrotal pain	*Bacteroides fragilis*, *Clostridium* spp, and *Klebsiella pneumonia*	Exploratory laparotomy with abdominoperineal resection and radical prostatectomy
Coulier et al. [9]	2004	67-year-old	Belgium	Diabetes	Diabetic patient presents with four days of left scrotal pain and swelling associated with discomfort in the left iliac fossa	NA	Hartmann surgical procedure associated with left scrotal exploration and drainage
Mathur et al. [10]	2011	52-year-old	India	Diabetes	Diabetic patient with a three-day history of pain and swelling in the left scrotum with fever	Escherichia coli	A left orchidectomy
Mandava et al. [11]	2014	51-year-old	India	Diabetes	Acute pain, redness, and swelling of the scrotum and fever of eight days duration. He was recently diagnosed with diabetes	NA	Intravenous antibiotics followed by right orchidectomy with surgical debridement
Lau et al. [12]	2018	80-year-old	Hong Kong	Hypertension, diabetes, and bullous pemphigoid	Hypertensive and diabetic patient with a two-day history of painful scrotal swelling and fever	Escherichia coli	Drainage of pus and left orchidectomy
Greear et al. [13]	2020	39-year-old	USA	T12 complete spinal cord injury neurogenic bladder, and chronic bacteriuria.	Four days after the hydrocelectomy the patient presented with recurrent scrotal swelling, fever and bilateral groin and lower abdominal discomfort	*Klebsiella pneumoniae* and *Enterococcus faecalis*	Drainage of serous fluid and right orchidectomy
Agrawal et al. [14]	2020	30-year-old	India	NA	Swelling in the right scrotum for 15 days associated with high-grade fever and pain in the scrotum	Escherichia coli	Incision and drainage

Discussion

Emphysematous epididymo-orchitis (EEO) is a rare but serious condition that is characterized by the development of gas-producing bacteria in the epididymis and/or testes. It is a subtype of necrotizing soft tissue infection, which is a rapidly progressing and potentially life-threatening condition that requires prompt diagnosis and treatment. EEO can lead to significant morbidity and mortality if not recognized and treated early [9,15].

Interestingly, the exact incidence of EEO is unknown, but it is believed to be a very rare condition, and only seven cases in the literature were reported. EEO is typically seen in men over the age of 50 with underlying comorbidities such as diabetes, immunosuppression, or urinary tract obstruction. This is related to the fact that diabetic patients have low immunity and impairing white blood cells to cover the site of infection as well as impaired microvascular circulation. However, our literature showed that most of the patients are old age or have comorbidities. Moreover, the most common causative organisms are *Escherichia coli* and *Klebsiella pneumoniae*, which are known to produce gas as a by-product of their metabolism. Other less common organisms include *Pseudomonas aeruginosa*, *Proteus mirabilis*, and anaerobes such as *Bacteroides fragilis* [13,16].

The pathophysiology of EEO is not completely understood, but it is believed to result from a combination of local tissue ischemia, impaired host immunity, and bacterial virulence factors. Gas-producing bacteria produce carbon dioxide, hydrogen, and nitrogen through the fermentation of glucose, which leads to a build-up of gas in the epididymis and/or testes. The gas produced by bacteria can cause tissue necrosis, inflammation, and abscess formation [12,17].

The clinical presentation of EEO is nonspecific and can include acute scrotal pain, swelling, erythema, and fever. Physical examination may reveal tenderness and swelling of the scrotum, and the affected testis may be indurated and tender to palpation. Because of that, the differential diagnosis of EEO includes testicular torsion, epididymitis, and testicular tumor [14,15].

Additionally, the diagnosis of EEO is usually made based on imaging studies such as ultrasound or computed tomography (CT) scan, which can show the presence of gas within the epididymis and/or testes. Laboratory studies may show leucocytosis and elevated inflammatory markers [2,18]

There is not much information available about the best modality for the treatment of this condition. Although, the literature suggests that the treatment of EEO involves aggressive management with antibiotics and surgical intervention. Early surgical exploration and debridement of the infected tissue are also recommended, along with broad-spectrum antibiotics that cover the most common causative organisms. In severe cases, orchiectomy may be necessary to prevent the spread of infection and sepsis [11,19].

The prognosis of EEO depends on the severity of the infection and the promptness of diagnosis and treatment. Delayed or inadequate treatment can lead to significant morbidity and mortality, including sepsis, multiorgan failure, and death. However, with early diagnosis and aggressive management, the prognosis of EEO is generally favorable [10,20]. Unfortunately, most of the patients in our review had an orchidectomy.

Limitations

The main limitation of this review was that it included only case reports that were not generalized. Moreover, a review article that included cohort studies could be better for detecting the exact presentation, management, and prognosis of EEO.

## Conclusions

Emphysematous epididymo-orchitis (EEO) is a rare but potentially life-threatening condition that requires prompt diagnosis and treatment. Although the exact pathophysiology of EEO is not fully understood, several risk factors have been identified, including diabetes mellitus and immunosuppression. Clinically, EEO can present with non-specific symptoms, making diagnosis challenging. Imaging studies such as ultrasound and CT scans can aid in the diagnosis of EEO and help guide appropriate management. Moreover, treatment typically involves surgical intervention, as well as antibiotic therapy and supportive care. Despite being a rare condition, EEO should be considered in the differential diagnosis of patients presenting with scrotal pain, swelling, and fever, particularly in those with underlying risk factors. Further research is needed to better understand the pathogenesis, risk factors, and optimal management of EEO.
